# Development and evaluation of a crosswalk between the SF-36 physical functioning scale and Health Assessment Questionnaire disability index in rheumatoid arthritis

**DOI:** 10.1186/1477-7525-11-199

**Published:** 2013-11-15

**Authors:** Peter M ten Klooster, Martijn AH Oude Voshaar, Barbara Gandek, Matthias Rose, Jakob B Bjorner, Erik Taal, Cees AW Glas, Piet LCM van Riel, Mart AFJ van de Laar

**Affiliations:** 1 Department of Psychology, Health and Technology, University of Twente, Enschede, The Netherlands; 2 Department of Quantitative Health Sciences, University of Massachusetts Medical School, Worcester, Massachusetts, USA; 3 Department of Psychosomatic Medicine, Charité University Medical Center, Berlin, Germany; 4 Department of Public Health, University of Copenhagen, Copenhagen, Denmark; 5 QualityMetric/OptumInsight, Lincoln, Rhode Island, USA; 6 Department of Research Methodology, Measurement and Data Analysis, University of Twente, Enschede, The Netherlands; 7 Department of Rheumatology, Radboud University Nijmegen Medical Centre, Nijmegen, The Netherlands; 8 Department of Rheumatology and Clinical Immunology, Medical Spectrum Twente, Enschede, The Netherlands

**Keywords:** Health assessment questionnaire, Item response theory, Linking, Physical functioning, Rheumatoid arthritis, Short form 36-item health survey (SF-36)

## Abstract

**Background:**

The SF-36 physical functioning scale (PF-10) and the Health Assessment Questionnaire disability index (HAQ-DI) are the most frequently used instruments for measuring self-reported physical function in rheumatoid arthritis (RA). The objective of this study was to develop a crosswalk between scores on the PF-10 and HAQ-DI in RA.

**Methods:**

Item response theory (IRT) methods were used to co-calibrate both scales using data from 1791 RA patients. The appropriateness of a Rasch-based crosswalk was evaluated by comparing it with crosswalks based on a two-parameter and a multi-dimensional IRT model. The accuracy of the final crosswalk was cross-validated using baseline (n = 532) and 6-month follow-up (n = 276) data from an independent cohort of early RA patients.

**Results:**

The PF-10 and HAQ-DI adequately fit a unidimensional Rasch model. Both scales measured a wide range of functioning, although the HAQ-DI tended to better target lower levels of functioning. The Rasch-based crosswalk performed similarly to crosswalks based on the two-parameter and multidimensional IRT models. Agreement between predicted and observed scale scores in the cross-validation sample was acceptable for group-level comparisons. The longitudinal validity in discriminating between disease response states was similar between observed and predicted scores.

**Conclusion:**

The crosswalk developed in this study allows for converting scores from one scale to the other and can be used for group-level analyses in patients with RA.

## Background

The SF-36 physical functioning scale (PF-10) [1,2] and the Health Assessment Questionnaire disability index (HAQ-DI) [3,4] are well-established instruments for measuring self-reported physical functioning. The SF-36 and the HAQ-DI were originally developed as generic measures to allow comparisons across populations [2,5]. but both instruments have also been thoroughly examined for use in several specific conditions, including rheumatoid arthritis (RA) [6].

Since the inclusion of patient-reported physical disability into core sets of outcomes for clinical trials and observational studies in RA [7,8], an increasing number of RA studies now assess and report physical functioning. Although variation in the choice of instrument exists, the HAQ-DI and PF-10 are among the most frequently used [9,10]. Both measures, however, differ considerably in their content, number of items, and scoring procedures, making it difficult to directly compare results obtained with the two scales. One way to overcome this problem is to link scores from the HAQ-DI and PF-10 [11]. This would allow the development of a concordance table, or crosswalk, to convert scores from one instrument to another and enable comparison of data from studies that used either one of the instruments.

Several methods are available for linking scale scores that vary in design, statistical techniques, and the degree to which exchangeability can be achieved [11,12]. Item response theory (IRT) offers a flexible and powerful framework for score linking by its inherent ability to calibrate different items of the same concept on a common underlying metric [13-16]. Several examples of how to use IRT modeling to develop crosswalks between different instruments intended to measure the same health domain have been reported [17-20]. IRT, however, makes certain assumptions about the nature of the data, in particular with respect to dimensionality. A variety of models are available, which differ in their restrictiveness with respect to the assumptions made and the number of parameters used to describe items [21]. Consequently, the type of linking and the accuracy of the resulting crosswalk may depend in part on the specific IRT model used.

The most basic form of IRT-based linking is possible when the responses on the two instruments follow the same Rasch model; that is, if it can be shown that they pertain to the same unidimensional latent trait and that all items are equally discriminating. In the Rasch model, the observed sum score is a sufficient statistic for the latent trait estimate [22]. If the Rasch model fits, linking boils down to estimating the trait level associated with an observed score on instrument A and then finding the observed score on instrument B associated with that trait level. In this approach, the statistical equating error is merely a function of the reliability of the two instruments, that is, the reliability with which trait levels can be estimated using either of the two instruments.

If the Rasch model does not fit, a more general model can be used such as a two-parameter IRT model that includes a discrimination parameter for differentially weighting the association of items with the latent variable. Although this extension may improve model fit, linking is less straightforward as the observed sum score is no longer a sufficient statistic for the trait level and, conditional on an observed sum score, estimates of trait levels vary to some degree. In this approach, an observed score on instrument A is associated with an expected trait level and from this expectation an expected observed score on instrument B is estimated. As such, the resulting crosswalk contains a second source of statistical error, attributable to the variation of the trait level given observed sum scores. This error, in turn, is a function of the magnitude of the discrimination indices, that is, the strength of the association of the items with the latent variable.

The linking approach can be further generalized by assuming that the two instruments measure two different, yet correlated latent variables. This situation can be modeled by a two-dimensional IRT model, where the responses on one instrument pertain to one latent variable, and the aggregation of the two latent variables has a two-dimensional normal distribution. Again, the observed sum score on instrument B is estimated from the observed score on instrument A via the IRT model. Added to the two sources of statistical error already identified, is an error associated with the magnitude of the correlation between the two latent variables, that is, the strength of the association between the two assumed latent scales.

To date, no studies have attempted to link PF-10 and HAQ-DI scores. Moreover, although many studies have reported high correlations between the instruments, the degree and consequences of the multidimensionality that would result from combining the scales are unclear. Some previous studies have suggested that the PF-10 and HAQ-DI, or a selection of its items used in the modified HAQ, do essentially measure the same concept [23,24]. However, studies that examined whether items from both scales could actually be calibrated on a common IRT metric did not unequivocally support either a unidimensional or multidimensional latent structure [25,26]. Moreover, these studies did not compare the performance of different IRT models to further examine the impact of multidimensionality.

This study presents the development and evaluation of a crosswalk between the PF-10 and the HAQ-DI in a large and clinically diverse sample of patients with RA who completed both instruments. The appropriateness of different IRT models is taken into account by comparing the calibrations and performance of a crosswalk based on a one-parameter Rasch model with those of its two-parameter and multidimensional extensions. The accuracy of the final crosswalk is cross-validated in an independent sample of patients with early RA participating in a treatment-to-target study.

## Methods

### Study populations

Two independent datasets were used for this study. The first dataset was used for IRT calibrations and development and comparison of the crosswalks between the PF-10 and the HAQ-DI. Accuracy and validity of the final crosswalk were tested in the second dataset.

#### Calibration sample

This dataset was derived from the Dutch Rheumatoid Arthritis Monitoring (DREAM) registry [27]. The DREAM registry is an observational multicenter cohort study that monitors the course of RA patients undergoing different treatment regimens in the Netherlands. Clinical, laboratory, and patient-reported outcomes are routinely collected and stored. Patient-reported outcomes are generally completed online. Within the different DREAM cohorts, 1791 unique patients simultaneously completed the SF-36 and HAQ-DI at least once between 2003 and 2012. For every patient, the first available simultaneous assessment was selected for analysis.

#### Cross-validation sample

The second, independent dataset included data from patients participating in the DREAM remission induction cohort [28]. The remission induction cohort consists of patients with early RA participating in a treat-to-target strategy aimed at achieving fast remission. The strategy has been shown to be highly effective, with the largest improvement in disease activity observed in the first 6 months of treatment [28]. Data from 532 patients who completed the HAQ-DI and SF-36 at baseline were used to cross-validate the accuracy of the Rasch-based crosswalk. To study the longitudinal performance of the crosswalk, available data of 276 patients who also completed the HAQ and PF-10 after six months were used.

### Measures

#### SF-36 physical functioning scale (PF-10)

The PF-10 is one of the eight scales of the SF-36 Health Survey and consists of 10 items measuring perceived current limitations in a variety of physical activities on a 3-point response scale from 1 (yes, limited a lot) to 3 (no, not limited at all). Where there are no missing data, observed PF-10 scores can have 21 possible values, with higher scores indicating more favorable levels of physical functioning. Using traditional scoring, scores on the PF-10 items are summed and linearly transformed to range between 0 and 100. Additionally, the summed scores can be standardized using norm-based scoring based on a mean score of 50 and a standard deviation of 10 in the 1998 US general population [29]. Previous Rasch modeling of the PF-10 indicated that the items form a unidimensional, hierarchical continuum with stable item difficulty estimates across diverse patient groups [24,30].

#### Health Assessment Questionnaire disability index (HAQ-DI)

The HAQ-DI contains 20 items measuring physical disabilities over the past week in eight categories of daily living: dressing and grooming, rising, eating, walking, hygiene, reach, grip, and activities. Each item is scored on a 4-point rating scale from 0 (without any difficulty) to 3 (unable to do). Additionally, the HAQ-DI contains four sections on the use of aids and devices or need for help from another person for performing activities in any of the eight categories. Two scoring methods can be used to calculate total HAQ-DI scores [31]. The standard disability index (SDI) adjusts category scores upwards for the use of aids or devices or help from others. The alternative disability index (ADI) does not take the use of aids and devices into account. For both scoring methods, the total disability score (HAQ-DI) is calculated by determining the highest score in each of the eight categories and then averaging the category scores. As a result, observed scores on the HAQ-DI can take on 25 possible values between 0 and 3, with higher values indicating more disability. Recent Rasch analyses have shown that the categories of the HAQ-DI constitute a unidimensional scale [24,32].

### Statistical analyses

#### IRT modeling

The maximum likelihood estimation procedure was utilized to estimate the structural model parameters and the latent disability levels of patients were estimated using the expected a posteriori (EAP) method throughout all IRT analyses. Model fit of all estimated models was assessed using Lagrange multiplier (LM) item fit statistics specifically targeted at polytomously scored items [33,34]. Absolute differences (effect sizes; ES) between expected and observed item scores for high, average and low scoring individuals were computed. In accordance with previous research, model fit was considered acceptable if all ES statistics were <0.10 [35,36]. As the ES is weighted by the number of response categories, this cutoff reflects differences between observed and expected score frequencies of 2.5% for the HAQ-DI and 3.33% for the PF-10, respectively. All IRT analyses were performed with the MIRT software package [37].

#### Development of the crosswalk

Initial IRT analysis and cross-calibration of the PF-10 and HAQ-DI were performed in the calibration sample. To achieve consistent response patterns, PF-10 scores were reversed (so a lower score indicates better function) preceding analysis. Item parameters for the Rasch-based crosswalk were obtained using the polytomous partial credit model (PCM) [38]. First, the 10 PF-10 items and the eight HAQ-DI category scores were jointly calibrated in the same model. After the structural model parameters were estimated, questionnaire-specific scoring runs on the HAQ-DI and PF-10 items only were performed to estimate EAP scores associated with all possible total score levels and to create scoring tables mapping this relation. In these runs the item parameters of the HAQ-DI and PF-10 items, respectively, were fixed to the values obtained in the initial co-calibration. Subsequently, each possible total score was linked to the total score on the other instrument for which the absolute distance between EAP scores on the latent scale was the smallest. The total procedure was separately performed for both the HAQ-SDI based category scores and the HAQ-ADI based category scores.

Next, the validity and appropriateness of the Rasch-based crosswalk was evaluated by determining its precision to correctly predict HAQ-DI scores from PF-10 scores and vice versa and comparing the results to the precision of two additional crosswalks that were developed using the two-parameter and multidimensional extensions of the PCM. Using the same general approach as outlined above, we first re-estimated the model parameters using the generalized partial credit model (GPCM). The GPCM model is a two-parameter IRT model for polytomous data which includes a discrimination parameter that accounts for the different reliability of individual items with respect to measuring the underlying latent trait. As such, the PCM is nested within the GPCM. Finally, a between-item, multidimensional GPCM model was estimated. Again, the GPCM model is nested within the multidimensional GPCM model. In this model, all items were specified to load on their own questionnaire-specific dimension, and the relation between the dimensions was modeled by their correlation. Because in this model the two dimension-specific EAP scores are estimated concurrently, no separate scoring runs needed to be performed for the HAQ-DI and PF-10 to obtain questionnaire-specific EAP estimates associated with all possible total scores.

Agreement between patients’ observed and predicted scores on the PF-10 and HAQ-DI was assessed by computing intraclass correlation coefficients (ICCs) with 95% confidence intervals using two-way mixed effects models with absolute agreement for single measurements (type A,1) [39]. ICCs were considered adequate for group level comparisons when ≥0.70 [40].

#### Cross-validation of the results

The final step of the analyses was to apply the crosswalk in the cross-validation sample and to evaluate the agreement between observed and predicted HAQ-DI and PF-10 scores. Agreement between patients’ observed and crosswalked scores on the PF-10 and HAQ-DI at baseline (n = 532) was again assessed by computing ICCs (type A,1). Additionally, Bland-Altman plots of the difference against the mean of predicted and observed scores were constructed [41,42]. As a final test of the validity of the crosswalk, observed and predicted change scores and total effect sizes (Cohen’s d) were calculated for patients who completed both measures at baseline and 6-month follow-up (n = 276). The relative efficiency of the change scores to discriminate between responder status was analyzed using one-way analysis of variance (ANOVA) tests [43,44]. The 28-joint Disease Activity Score (DAS28), a pooled index that includes a tender joint count, a swollen joint count, the erythrocyte sedimentation rate, and the patient’s global assessment of general health, was used as the external criterion for determining response to treatment [45]. Patients were classified as good responders at 6 months when the DAS28 score had improved at least 1.2 points and the final score was ≤3.2 [46]. For purposes of comparing results, relative validity (RV) coefficients with 95% bias-corrected and accelerated bootstrap confidence intervals [44,47] for the predicted scores in relation to the actual observed scores were computed.

## Results

### Patient characteristics

The calibration and cross-validation samples were comparable with respect to demographic characteristics (Table 1). However, baseline physical functioning levels were substantially better in the cross-validation sample, as measured with both the HAQ-DI and the PF-10. Patients in the cross-validation sample had moderately active disease on average at baseline according to the DAS28.

**Table 1:** Patient characteristics

	**Calibration**	**Cross-validation**
	**sample**	**sample**
	**(n = 1791)**	**(n = 532)**
Sex, % female	69.2	63.0
Age in years, mean (SD)	56.54 (13.31)	56.48 (14.26)
HAQ-SDI (0–3), mean (SD)*	1.08 (0.71)	0.65 (0.65)
PF-10 (0–100), mean (SD)	53.89 (26.35)	67.39 (25.71)
DAS28, mean (SD)*	-	4.28 (1.51)
VAS Pain (0–100), mean (SD)*	-	43.38 (26.23)
VAS General Health (0–100), mean (SD)*	-	44.49 (26.48)

HAQ-SDI = Health Assessment Questionnaire standard disability index; PF-10 = SF-36 physical functioning scale; DAS28 = 28-joint Disease Activity Score; VAS Pain = visual analog scale for patient’s pain in the past week; VAS General Health = visual analog scale for patient’s general health in the past week. * Higher values indicate worse health states.

### Development of the Rasch-based crosswalk

Total scores on the PF-10 and HAQ-DI were strongly correlated (r = −0.75 for both the HAQ-SDI and HAQ-ADI). Both the Rasch-based co-calibration of the HAQ-SDI and PF-10 items and the co-calibration of HAQ-ADI and PF-10 items resulted in a model that adequately fitted the data according to the LM tests, with all accompanying ESs <0.10 (Additional file 1: Table S1 and S2).

**Figure 1:**
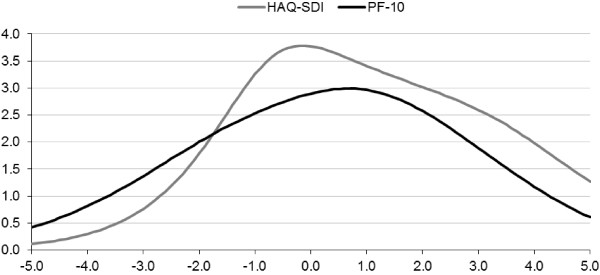
**Test information function curve (partial credit model) for the HAQ-SDI and PF-10 in relation to theta.** The test information function is the sum of all separate item information functions. Higher positive theta scores indicate worse physical functioning.

Figure 1 presents the test information functions which describe the local reliability of the PF-10 and HAQ-SDI. Both scales measured an approximately equally wide range of physical functioning with high precision. Overall, the PF-10 was slightly more precise at better levels of physical functioning (i.e., lower theta values), whereas the HAQ-SDI tended to provide more information at worse levels of functioning.

Table 2 presents the resulting Rasch-based crosswalks between the PF-10 and HAQ-DI. Separate cross-walks are presented for the standard and alternative scoring rule of the HAQ-DI. As would be expected, predicted HAQ-ADI scores were generally lower than predicted HAQ-SDI scores, for a given level of the PF-10. This effect was strongest in the range of HAQ-DI scores from 1 to 2, where for the same observed PF-10 total scores, the estimated HAQ-SDI scores were consistently 0.25 points (i.e. two score levels) higher than the HAQ-ADI scores. Observed HAQ-ADI and HAQ-SDI scores ≥2.75 were linked to locations on the EAP theta scale that were below the lowest possible score for the PF-10 scale. Conversely, observed PF-10 scores of 95 and 100 were linked to EAP scores that reflect levels of function that are not represented in the HAQ-DI. They were therefore linked to the value zero in the crosswalks.

**Table 2:** Rasch-based crosswalk for transforming PF-10 scores into HAQ-DI scores and vice versa

**HAQ standard scoring (with aids and devices)**				**HAQ alternative scoring (without aids and devices)**			
**Observed**	**Predicted**	**Observed**	**Predicted**	**Observed**	**Predicted**	**Observed**	**Predicted**
**HAQ-SDI**	**PF-10**	**PF-10**	**HAQ-SDI**	**HAQ-ADI**	**PF-10**	**PF-10**	**HAQ-ADI**
**score**	score*	score*	**score**	**score**	score*	score*	**score**
0.000	95 (54.9)	100 (57.0)	0.000	0.000	95 (54.9)	100 (57.0)	0.000
0.125	90 (52.8)	95 (54.9)	0.000	0.125	85 (50.7)	95 (54.9)	0.000
0.250	85 (50.7)	90 (52.8)	0.125	0.250	80 (48.6)	90 (52.8)	0.125
0.375	75 (46.5)	85 (50.7)	0.250	0.375	75 (46.5)	85 (50.7)	0.125
0.500	75 (46.5)	80 (48.6)	0.250	0.500	70 (44.4)	80 (48.6)	0.250
0.625	70 (44.4)	75 (46.5)	0.375	0.625	65 (42.3)	75 (46.5)	0.375
0.750	65 (42.3)	70 (44.4)	0.625	0.750	55 (38.1)	70 (44.4)	0.500
0.875	60 (40.2)	65 (42.3)	0.750	0.875	50 (36.0)	65 (42.3)	0.625
1.000	55 (38.1)	60 (40.2)	0.875	1.000	45 (33.9)	60 (40.2)	0.625
1.125	50 (36.0)	55 (38.1)	1.000	1.125	45 (33.9)	55 (38.1)	0.750
1.250	45 (33.9)	50 (36.0)	1.125	1.250	40 (31.8)	50 (36.0)	1.000
1.375	45 (33.9)	45 (33.9)	1.375	1.375	35 (29.7)	45 (33.9)	1.125
1.500	40 (31.8)	40 (31.8)	1.500	1.500	30 (27.6)	40 (31.8)	1.250
1.625	35 (29.7)	35 (29.7)	1.625	1.625	25 (25.5)	35 (29.7)	1.375
1.750	30 (27.6)	30 (27.6)	1.750	1.750	25 (25.5)	30 (27.6)	1.500
1.875	25 (25.5)	25 (25.5)	1.875	1.875	25 (25.5)	25 (25.5)	1.750
2.000	20 (23.4)	20 (23.4)	2.125	2.000	20 (23.4)	20 (23.4)	2.000
2.125	20 (23.4)	15 (21.3)	2.250	2.125	15 (21.3)	15 (21.3)	2.125
2.250	15 (21.3)	10 (19.2)	2.375	2.250	10 (19.2)	10 (19.2)	2.250
2.375	10 (19.2)	5 (17.0)	2.625	2.375	10 (19.2)	5 (17.0)	2.625
2.500	5 (17.0)	0 (14.9)	2.750	2.500	5 (17.0)	0 (14.9)	2.750
2.625	5 (17.0)			2.625	5 (17.0)		
2.750	0 (14.9)			2.750	0 (14.9)		
2.875	0 (14.9)			2.875	0 (14.9)		
3.000	0 (14.9)			3.000	0 (14.9)		

*Scores are original 0–100 transformed scale scores, 1998 US norm-based T-scores are presented between brackets.

### Comparative performance of the Rasch-based crosswalk

Model fit of the co-calibrations based on the two-parameter GPCM and the multi-dimensional IRT model improved marginally as compared with the Rasch model (Additional file 1: Table S3–S6). For both the GPCM and the multi-dimensional model, ESs were also <0.10 and generally slightly smaller than those observed in the Rasch model. The correlation between the latent dimensions in the multidimensional models was 0.73. The crosswalks based on the GPCM and multi-dimensional IRT model were almost identical to the Rasch-based crosswalk. Correlations between predicted scores based on the different crosswalks were very high (r’s >0.988). Moreover, the crosswalks based on the two-parameter and multidimensional models did not perform substantially better in terms of agreement between observed and predicted total scores on the PF-10 and HAQ-DI (Table 3). Considering that the Rasch-based calibration fitted the data well according to pre-specified criteria and that the agreement between observed and predicted scale scores did not improve much in the more general models, it was concluded that the Rasch-based crosswalk was adequate for converting total scale scores.

**Table 3:** Agreement (ICC, 95% CI) between observed and predicted total scale scores using crosswalks based on the different IRT models in the calibration sample (n = 1791)

	**Rasch**	**Two-parameter**	**Multi-dimensional**
	**model**	**model**	**model**
HAQ-SDI	0.739 (0.717 to 0.760)	0.741 (0.719 to 0.762)	0.739 (0.717 to 0.760)
HAQ-ADI	0.737 (0.714 to 0.758)	0.737 (0.715 to 0.758)	0.735 (0.712 to 0.756)
PF-10 (predicted from HAQ-SDI)	0.746 (0.724 to 0.767)	0.745 (0.722 to 0.765)	0.742 (0.720 to 0.763)
PF-10 (predicted from HAQ-ADI)	0.748 (0.726 to 0.768)	0.750 (0.728 to 0.770)	0.749 (0.727 to 0.769)

ICC = intraclass correlation coefficient; HAQ-SDI = Health Assessment Questionnaire standard disability index; HAQ-ADI = Health Assessment Questionnaire alternative disability index; PF-10 = SF-36 physical functioning scale.

### Cross-validation of the results

**Figure 2:**
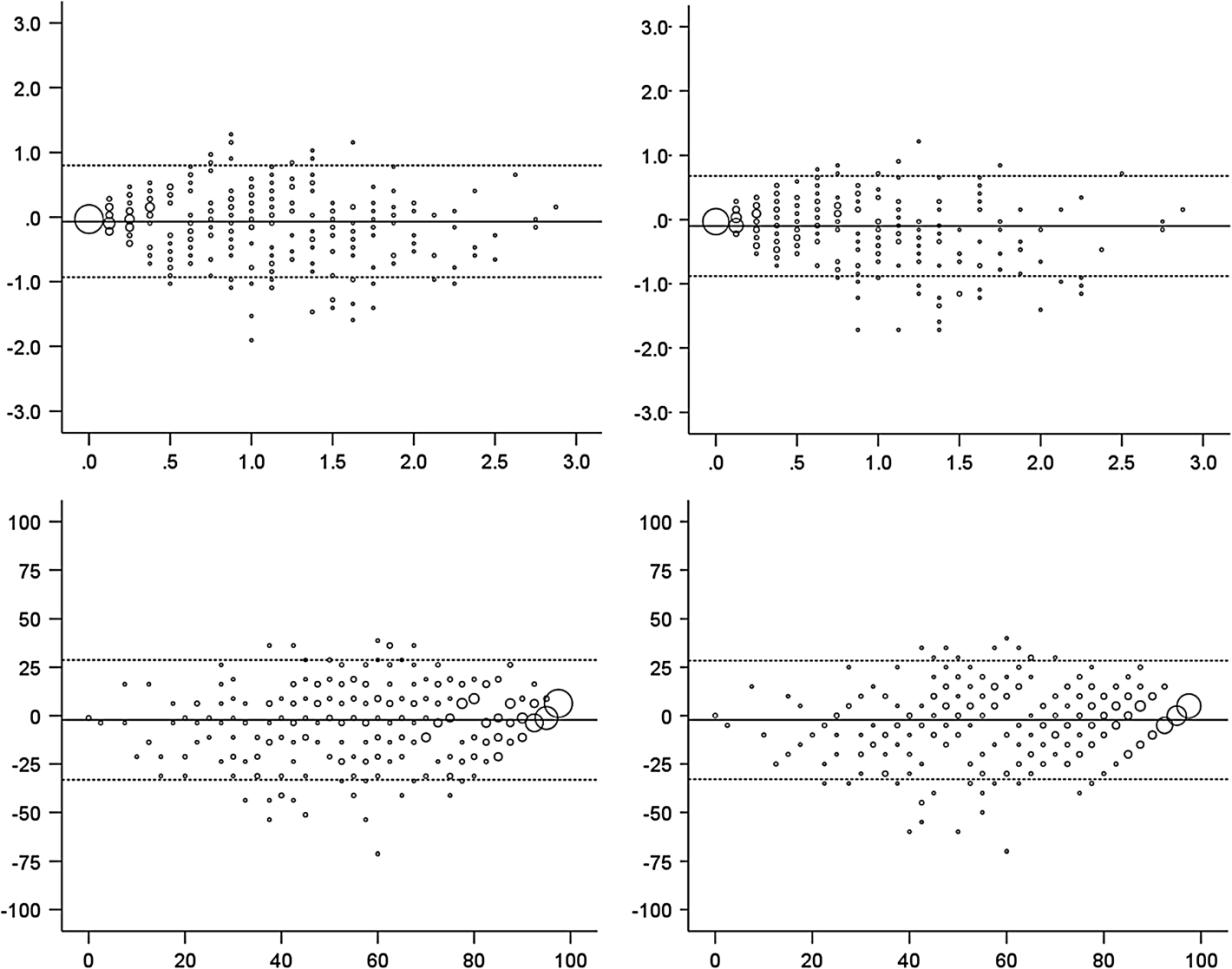
**Bland-Altman plots for agreement between observed and predicted HAQ-SDI (top left), HAQ-ADI (top right) and PF-10 scores (bottom left: predicted from HAQ-SDI, bottom right: predicted from HAQ-ADI).** The y-axes represent the difference between observed and predicted scores. The x-axes represent the mean of observed and predicted scores. The horizontal solid lines represent the mean difference between both scores. The dashed lines represent the 95% limits of agreement.

The agreement between observed scores and scores predicted from the Rasch-based crosswalk was high in the cross-validation sample. The ICCs (95% CI) between predicted and actual scores were 0.78 (0.74 to 0.81) for the HAQ-SDI, 0.77 (0.72 to 0.80) for the HAQ-ADI and 0.79 (0.75 to 0.82) for the PF-10, indicating adequate agreement for group-level comparisons. Additionally, group mean differences on both scales were small in magnitude (Table 4). Intra-individual differences were similarly distributed above and below the mean and not related to the magnitude of the measurement (Figure 2). However, the limits of agreement were wide for both scales and showed substantial discrepancies in agreement within individual patients.

**Table 4:** Agreement between observed and predicted scores on the HAQ-DI and PF-10 in the cross-validation sample (n = 532)

	**ICC (95% CI)**	**Mean (SD)**	**Mean (SD)**	**Mean (SD)**	**LOA**
		**observed scores**	**predicted scores**	**difference**	
HAQ-SDI	0.78 (0.74 to 0.81)	0.65 (0.64)	0.72 (0.71)	−0.07 (0.44)	−0.93 to 0.80
HAQ-ADI	0.77 (0.72 to 0.80)	0.53 (0.57)	0.63 (0.65)	−0.10 (0.40)	−0.88 to 0.68
PF-10 (predicted from HAQ-SDI)	0.79 (0.75 to 0.82)	67.39 (25.71)	69.60 (23.26)	−2.21 (15.80)	−33.18 to 28.76
PF-10 (predicted from HAQ-ADI)	0.79 (0.76 to 0.82)	67.39 (25.71)	69.62 (23.01)	−2.23 (15.64)	−32.88 to 28.42

HAQ-DI = Health Assessment Questionnaire Disability Index; PF-10 = SF-36 physical functioning scale; ICC = intraclass correlation coefficient; LOA = Bland-Altman limits of agreement (mean difference ± 1.96×SD of the difference).

With respect to the observed 6-month change scores in the total cross-validation sample (Table 5), standardized improvements were largest for the HAQ-DI (ES = 0.55), closely followed by the HAQ-SDI (ES = 0.49) and the PF-10 (ES = 0.40). In terms of differentiating between levels of longitudinal treatment response, the HAQ-ADI was slightly more efficient than the HAQ-SDI and PF-10. Relative validity coefficients of the predicted scores were close to, and not significantly different from, those of the actual observed scores for all three scales.

**Table 5:** Baseline to 6-month effect sizes and mean (SD) changes in physical functioning scores across levels of DAS28 treatment response in the cross-validation sample (n = 276)

	**Total ES**	**Non- or moderate responders**	**Good responders**	***F***	**RV**	**95% CI**
	** **	**(n = 142)**	**(n = 134)**			
HAQ-ADI						
Observed	0.55	−0.20 (0.52)	−0.62 (0.58)	38.59	1.00	
Predicted	0.37	−0.14 (0.54)	−0.50 (0.55)	30.12	0.78	0.35 to 1.69
HAQ-SDI						
Observed	0.49	−0.17 (0.44)	−0.49 (0.49)	30.63	1.00	
Predicted	0.38	−0.14 (0.53)	−0.51 (0.55)	32.38	1.06	0.44 to 2.25
PF-10						
Observed	0.40	5.53 (20.15)	19.63 (20.74)	32.80	1.00	
Predicted from HAQ-ADI	0.55	7.85 (19.99)	24.33 (21.98)	42.52	1.18	0.63 to 3.35
Predicted from HAQ-SDI	0.48	6.23 (15.34)	16.98 (16.68)	31.09	0.86	0.43 to 2.36

PF-10 = SF-36 physical functioning scale; HAQ-SDI = Health Assessment Questionnaire standard disability index; HAQ-ADI = Health Assessment Questionnaire alternative disability index; ES = Cohen’s *d* effect size; *F* = F-statistic from one-way ANOVA; RV = Relative validity (ratio of F-statistics compared with observed score); 95% CI = 95% bootstrap bias-corrected and accelerated confidence interval.

## Discussion

This study used IRT methods to analyze and link two widely used scales for measuring physical functioning, the PF-10 and the HAQ-DI. Results showed that it was possible to develop a straightforward Rasch-based crosswalk between both scales that can be used to estimate scores on one scale from scores on the other in patients with RA. The Rasch-based crosswalk performed similarly to crosswalks based on its two-parameter and multidimensional extensions. The application of the crosswalk in an independent sample of patients with early RA indicated that the crosswalk can be validly used for group-level analyses in RA populations.

Test linking or test equating has long been the focus of research in educational and psychological settings [12,48]. More recently, the desire for standardization has also found its way to health outcomes measurement. As in educational testing, linking of existing health outcome instruments could enhance meaningful comparison and interpretation of results across studies and populations. With the rise of IRT in health outcomes assessment, new techniques have become available to achieve this objective. This is reflected in an increasing number of studies that have linked different patient-reported measures using IRT-based methods, including several measures of physical functioning [15,17,19,49-55]. These crosswalks allow researchers to compare their results with studies and populations where another instrument was used and may improve the common understanding of the specific underlying construct. Moreover, they may be particularly useful for compilation of findings in meta-analytic studies or longitudinal studies focusing on measuring effects or changes [56]. A such, crosswalks are an important step in achieving better interpretation and comparability of patient-reported outcomes measures across different studies [57]. A next possible step in the standardization and promotion of a common measurement system of patient-reported outcomes, is the development of large IRT-calibrated item banks such as those developed by the Patient-Reported Outcomes Measurement Information System (PROMIS) initiative [58]. These item banks can be used to build flexible short forms and computer adaptive tests for different populations or clinical conditions, while scores on these measures remain directly comparable. Recent studies have already shown the promise of this approach in RA [59].

The current study used an elaborate approach for cross-calibrating the HAQ-DI with the PF-10 and developing and evaluating the crosswalk, especially in its choice for comparing different IRT models. IRT linking studies usually do not explain or justify their use of a specific IRT model, such as the Rasch model or more general models. When using IRT analysis, however, the differences in model assumptions should be taken into account and the final model choice should be motivated by considering aspects such as the unidimensionality and the discrimination equality of the items [60]. Moreover, it should be shown to what degree the used model holds. In the case of using IRT for linking total scale scores, the specific model used may have consequences for the robustness and accuracy of the resulting crosswalk. This article presents a straightforward and practical IRT-based approach of linking total scale scores that includes comparing the fit and performance of different nested IRT models. This approach can be used for future studies aimed at linking different instruments intended to measure the same construct. An important feature of the approach is that it can be used for calibrating scales with polytomous items, which is the case with most patient-reported outcomes. Contrary to the Rasch model, tests of model fit for more complex models for polytomous items which are based on test statistics with known asymptotic distributions are rare. Therefore, the presented approach uses the LM test throughout all fit analyses [34].

Additionally, most IRT linking studies to date have not tested the performance of the crosswalks in clinically different, independent samples. To our knowledge, this study is the first to cross-validate a crosswalk of physical functioning scales in a clinical setting. One recent study did validate a crosswalk for fatigue using data from a subsequent time point, but acknowledged that using an independent sample would have been preferable [56]. With the objective in mind of creating a robust crosswalk in this study, its development was performed in a large and diverse sample of RA patients with a wide range of physical functioning levels. Subsequently, the performance of the crosswalk was examined in a specific sample of patients with early disease.

The results of the IRT calibrations suggested that the PF-10 and the HAQ-DI essentially measure the same unidimensional construct and could be adequately fitted to the same Rasch model. The finding that the simple Rasch model performed similarly to more general models in calibrating both scales may have several theoretical and practical advantages [61-63]. An advantage in the case of total score linking is that each observed total instrument score is associated with only one latent trait (theta) score, making the resulting crosswalk more straightforward and robust against statistical error.

The evaluation of the measurement precision of the PF-10 and HAQ-DI under the Rasch model showed that the HAQ-DI and the PF-10 both measured a wide range of physical functioning in patients with RA. However, the HAQ-DI provided its optimal measurement precision at worse levels of physical function, whereas the PF-10 had better precision at somewhat better levels on the physical function continuum. This corresponds with previously reported ceiling effects of the HAQ-DI in less disabled populations [24,64-66] and floor effects of the PF-10 in more disabled populations [67-70]. These effects were also apparent in the final crosswalk, where the HAQ-DI was better able to distinguish different scores at the lower end of the physical functioning spectrum and the PF-10 could better distinguish scores at the upper end. This supports previous findings that combining items from the HAQ-DI and PF-10 can reduce floor and ceiling effects and results in a scale with increased measurement precision and sensitivity to change across a wider range of physical functioning [25].

In the current study, separate crosswalks were developed for so-called standard (SDI) and alternative disability index (ADI) scoring of the HAQ-DI [5]. In the standard scoring method, the score on a category of daily living is corrected upwards when a respondent indicates the use of help from others or a device for performing one of the items in this category. Consequently, SDI scores are generally higher than ADI scores. Although the average difference between both scoring methods has been reported to be very small in general populations or populations with mild disability [71], SDI scores have been shown to be up to 0.15 to 0.26 points higher than ADI scores in samples with increasing disability levels [65,72-74]. In the current study, this resulted in higher predicted scores for the SDI than for the ADI, especially for patients with worse levels of functioning. Therefore, care must be taken in using the correct crosswalk when converting PF-10 and HAQ-DI scores. Unfortunately, published studies do not always clearly specify which method was used to compute the HAQ-DI scores [75,76]. If necessary and possible, researchers should therefore re-analyze the original data to compute the correct HAQ-DI scores.

Additionally, we presented the cross-walk for both the original and the norm-based scoring method of the PF-10. The original 0–100 scoring has been most frequently used in the literature to date. Since the introduction of version 2 of the SF-36, however, all eight scales can also be linearly transformed to T-scores based on normative data from the US general population [29]. This norm-based scoring method has become increasingly popular as it allows for easier interpretation of differences across scales and populations.

The two RA samples used to develop and evaluate the crosswalk in this study correspond with the two major populations of interest in current clinical studies in RA. The sample used to cross-calibrate the PF-10 and HAQ-DI represents the general and clinically diverse RA population seen in the everyday clinical practice and the distribution of age, sex, and functional disability scores in this sample corresponds closely with the characteristics reported in other large observational studies [77-79]. The cross-validation was performed in a sample of RA patients with a maximum symptom duration of one year. This population is gaining increasing research interest, mainly due to the development of effective biological treatments and the implementation of new treatment guidelines [80,81]. The finding that the crosswalk also performed well in this very specific sample, provides further support for its wide applicability in RA research.

It should be noted, however, that RA is characterized by very specific disease mechanisms and physical manifestations, such as a high frequency of dexterity problems. Consequently, the IRT item parameters of the HAQ-DI and PF-10 may vary between conditions and populations as was previously shown for the HAQ-DI across different rheumatic diseases [35]. Therefore, future studies should cross-validate the crosswalk in both general and other disease-specific populations.

Further, the crosswalk is not suitable for use at the individual patient level. Although ICCs between observed and predicted scores were adequate for group-level analyses, they were not sufficiently high to warrant individual level analyses. This was confirmed by the Bland-Altman analyses, which showed that observed and predicted scores were characterized by high intra-individual variation. Therefore, cross-walked scores are not equivalent at an individual level and cannot be used interchangeably.

## Conclusions

In sum, the crosswalk developed in this study enables the conversion of PF-10 scores into HAQ-DI scores and vice versa in patients with RA. Using the crosswalk will allow for group-level comparisons of data from studies that used either of the scales and can facilitate more meaningful interpretation and comparison of results. Future studies should examine the robustness of the crosswalk in other populations.

## Abbreviations

ADI: Alternative disability index; DAS28: 28-joint disease activity score; DREAM: Dutch rheumatoid arthritis monitoring; EAP: Expected a posteriori; ES: Effect size; GPCM: Generalized partial credit model; HAQ-DI: Health assessment questionnaire disability index; ICC: Intraclass correlation coefficient; IRT: Item response theory; LM: Lagrange multiplier; PCM: Partial credit model; PF-10: 10-item physical functioning scale; RA: Rheumatoid arthritis; RV: Relative validity; SDI: Standard disability index; SF-36: Short-form 36-item health survey.

## Competing interests

The authors declare that they have no competing interests.

## Authors’ contributions

PTK and MOV designed the study and drafted the manuscript. MOV and CG carried out the statistical analyses. BG, MR, JB, ET, PVR and MVDL supervised the study and the interpretation of the results. All authors critically reviewed, contributed to and approved the final manuscript.

## Supplementary Material

Additional file 1: Table S1 Item parameters and item level fit statistics for the Rasch (PCM) co-calibration of the HAQ-SDI and PF-10. **Table S2**. Item parameters and item level fit statistics for the Rasch (PCM) co-calibration of the HAQ-ADI and PF-10. **Table S3**. Item parameters and item level fit statistics for the two-parameter (GPCM) co-calibration of the HAQ-SDI and PF-10. **Table S4**. Item parameters and item level fit statistics for the two-parameter (GPCM) co-calibration of the HAQ-ADI and PF-10. **Table S5**. Item parameters and item level fit statistics for the multidimensional (GPCM) co-calibration of the HAQ-SDI and PF-10. **Table S6**. Item parameters and item level fit statistics for the multidimensional (GPCM) co-calibration of the HAQ-ADI and PF-10. Click here for file

## References

[B1] Stewart AL Kamberg CJ Stewart AL, Ware JEJr Physical functioning measures Measuring functioning and well-being 1992 Durham, NC: Duke University Press 86 101

[B2] Ware JE Jr Sherbourne CD The MOS 36-item short-form health survey (SF-36). I. Conceptual framework and item selection Med Care 1992 30 473 483 10.1097/00005650-199206000-00002 1593914

[B3] Fries JF Spitz P Kraines RG Holman HR Measurement of patient outcome in arthritis Arthritis Rheum 1980 23 137 145 10.1002/art.1780230202 7362664

[B4] Fries JF Spitz PW Young DY The dimensions of health outcomes: the Health Assessment Questionnaire, disability and pain scales J Rheumatol 1982 9 789 793 7175852

[B5] Bruce B Fries JF The Health Assessment Questionnaire (HAQ) Clin Exp Rheumatol 2005 23 S14 S18 16273780

[B6] Oude Voshaar MA ten Klooster PM Taal E van de Laar MA Measurement properties of physical function scales validated for use in patients with rheumatoid arthritis: a systematic review of the literature Health Qual Life Outcomes 2011 9 99 10.1186/1477-7525-9-99 22059801 PMC3221621

[B7] Boers M Tugwell P Felson DT van Riel PL Kirwan JR Edmonds JP Smolen JS Khaltaev N Muirden KD World health organization and international league of associations for rheumatology core endpoints for symptom modifying antirheumatic drugs in rheumatoid arthritis clinical trials J Rheumatol 1994 21 86 89 7799394

[B8] Wolfe F Lassere M van der Heijde D Stucki G Suarez-Almazor M Pincus T Eberhardt K Kvien TK Symmons D Silman A Preliminary core set of domains and reporting requirements for longitudinal observational studies in rheumatology J Rheumatol 1999 26 484 489 9972992

[B9] Kalyoncu U Dougados M Daures JP Gossec L Reporting of patient-reported outcomes in recent trials in rheumatoid arthritis: a systematic literature review Ann Rheum Dis 2009 68 183 190 10.1136/ard.2007.084848 18375533

[B10] Kirkham JJ Boers M Tugwell P Clarke M Williamson PR Outcome measures in rheumatoid arthritis randomised trials over the last 50 years Trials 2013 14 324 10.1186/1745-6215-14-324 24103529 PMC3852710

[B11] Dorans NJ Linking scores from multiple health outcome instruments Qual Life Res 2007 16 85 94 10.1007/s11136-006-9155-3 17286198

[B12] Lim RL Linking results of distinct assessments Appl Meas Educ 1993 6 83 102 10.1207/s15324818ame0601_5

[B13] Chang CH Reeve BB Item response theory and its applications to patient-reported outcomes measurement Eval Health Prof 2005 28 264 282 10.1177/0163278705278275 16123257

[B14] McHorney CA Generic health measurement: past accomplishments and a measurement paradigm for the 21st century Ann Intern Med 1997 127 743 750 10.7326/0003-4819-127-8_Part_2-199710151-00061 9382391

[B15] McHorney CA Cohen AS Equating health status measures with item response theory: illustrations with functional status items Med Care 2000 38 II43 II59 10982089 10.1097/00005650-200009002-00008

[B16] Reise SP Waller NG Item response theory and clinical measurement Annu Rev Clin Psychol 2009 5 27 48 10.1146/annurev.clinpsy.032408.153553 18976138

[B17] Fisher WP Jr Eubanks RL Marier RL Equating the MOS SF36 and the LSU HSI physical functioning scales J Outcome Meas 1997 1 329 362 9661727

[B18] Orlando M Sherbourne CD Thissen D Summed-score linking using item response theory: application to depression measurement Psychol Assess 2000 12 354 359 11021160 10.1037//1040-3590.12.3.354

[B19] Carmody TJ Rush AJ Bernstein I Warden D Brannan S Burnham D Woo A Trivedi MH The Montgomery Asberg and the Hamilton ratings of depression: a comparison of measures Eur Neuropsychopharmacol 2006 16 601 611 10.1016/j.euroneuro.2006.04.008 16769204 PMC2151980

[B20] Fischer HF Tritt K Klapp BF Fliege H How to compare scores from different depression scales: equating the patient health questionnaire (PHQ) and the ICD-10-symptom rating (ISR) using item response theory Int J Methods Psychiatr Res 2011 20 203 214 10.1002/mpr.350 22021205 PMC6878401

[B21] Hambleton RK Swaminathan H Rogers HJ Fundamentals of item response theory 1991 Newbury Park, CA: Sage

[B22] Andersen EB Sufficient statistics and latent trait models Psychometrika 1977 42 69 81 10.1007/BF02293746

[B23] Escalante A Del RI Cornell JE Latent variable approach to the measurement of physical disability in rheumatoid arthritis Arthritis Rheum 2004 51 399 407 10.1002/art.20404 15188325

[B24] Taylor WJ McPherson KM Using Rasch analysis to compare the psychometric properties of the Short Form 36 physical function score and the health assessment questionnaire disability index in patients with psoriatic arthritis and rheumatoid arthritis Arthritis Rheum 2007 57 723 729 10.1002/art.22770 17530670

[B25] Martin M Kosinski M Bjorner JB Ware JE Jr Maclean R Li T Item response theory methods can improve the measurement of physical function by combining the modified health assessment questionnaire and the SF-36 physical function scale Qual Life Res 2007 16 647 660 10.1007/s11136-007-9193-5 17334829

[B26] Rose M Bjorner JB Becker J Fries JF Ware JE Evaluation of a preliminary physical function item bank supported the expected advantages of the Patient-Reported Outcomes Measurement Information System (PROMIS) J Clin Epidemiol 2008 61 17 33 10.1016/j.jclinepi.2006.06.025 18083459

[B27] Kievit W Fransen J Oerlemans AJ Kuper HH van de Laar MA de Rooij DR De Gendt CM Ronday KH Jansen TL van Oijen PC The efficacy of anti-TNF in rheumatoid arthritis, a comparison between randomized controlled trials and clinical practice Ann Rheum Dis 2007 66 1473 1478 10.1136/ard.2007.072447 17426065 PMC2111629

[B28] Vermeer M Kuper HH Hoekstra M Haagsma CJ Posthumus MD Brus HL van Riel PL van de Laar MA Implementation of a treat-to-target strategy in very early rheumatoid arthritis: results of the Dutch rheumatoid arthritis monitoring remission induction cohort study Arthritis Rheum 2011 63 2865 2872 10.1002/art.30494 21647867

[B29] Ware JE Kosinski M Dewey JE How to score version 2 of the SF-36 health survey (standard & acute forms) 2000 QualityMetric Inc: Lincoln, RI

[B30] Haley SM McHorney CA Ware JE Jr Evaluation of the MOS SF-36 physical functioning scale (PF-10): I. Unidimensionality and reproducibility of the Rasch item scale J Clin Epidemiol 1994 47 671 684 10.1016/0895-4356(94)90215-1 7722580

[B31] Fries JF The Health Assessment Questionnaire (HAQ) and the Improved HAQ 2009 Stanford: Stanford University School of Medicine, Division of Immunology & Rheumatology Available at: http://aramis.stanford.edu/haq.html

[B32] ten Klooster PM Taal E van de Laar MA Rasch analysis of the Dutch health assessment questionnaire disability index and the health assessment questionnaire II in patients with rheumatoid arthritis Arthritis Rheum 2008 59 1721 1728 10.1002/art.24065 19035413

[B33] Glas CAW Modification indices for the 2-PL and the nominal response model Psychometrika 1999 64 273 294 10.1007/BF02294296

[B34] Glas CAW Nering ML, Ostini R Testing fit to IRT models for polytomously scored items Handbook of polytomous item response theory models 2010 New York, NY: Routledge 185 210

[B35] van Groen MM ten Klooster PM Taal E van de Laar MA Glas CA Application of the health assessment questionnaire disability index to various rheumatic diseases Qual Life Res 2010 19 1255 1263 10.1007/s11136-010-9690-9 20559736 PMC2963741

[B36] Oude Voshaar MA Glas CA ten Klooster PM Taal E Wolfe F van de Laar MA Cross-cultural measurement equivalence of the health assessment questionnaire-II Arthritis Care Res (Hoboken) 2012 65 1000 1004 23225345 10.1002/acr.21919

[B37] Glas CA Preliminary manual of the software program Multidimensional Item Response Theory (MIRT) 2010 Enschede, The Netherlands: University of Twente Available at: http://www.utwente.nl/gw/omd/en/employees/employees/glas.doc/

[B38] Masters GN Wright BD van der Linden WJ, Hambleton RK The partial credit model Handbook of modern item response theory 1997 New York: Springer 101 122

[B39] McGraw KO Wong SP Forming inferences about some intraclass correlation coefficients Psychol Methods 1996 1 30 46

[B40] Lohr KN Assessing health status and quality-of-life instruments: attributes and review criteria Qual Life Res 2002 11 193 205 10.1023/A:1015291021312 12074258

[B41] Bland JM Altman DG Statistical methods for assessing agreement between two methods of clinical measurement Lancet 1986 i 307 310 2868172

[B42] Bland JM Altman DG Comparing methods of measurement: why plotting difference against standard method is misleading Lancet 1995 346 1085 1087 10.1016/S0140-6736(95)91748-9 7564793

[B43] McHorney CA Ware JE Jr Raczek AE The MOS 36-Item short-form health survey (SF-36): II. Psychometric and clinical tests of validity in measuring physical and mental health constructs Med Care 1993 31 247 263 10.1097/00005650-199303000-00006 8450681

[B44] Liang MH Larson MG Cullen KE Schwartz JA Comparative measurement efficiency and sensitivity of five health status instruments for arthritis research Arthritis Rheum 1985 28 542 547 10.1002/art.1780280513 4004963

[B45] Prevoo ML Hof MA V ’t Kuper HH Van Leeuwen MA Van De Putte LB Van Riel PL Modified disease activity scores that include twenty-eight-joint counts: development and validation in a prospective longitudinal study of patients with rheumatoid arthritis Arthritis Rheum 1995 38 44 48 10.1002/art.1780380107 7818570

[B46] Fransen J van Riel PL The disease activity score and the EULAR response criteria Clin Exp Rheumatol 2005 23 S93 S99 16273792

[B47] Deng N Ware J Using bootstrap confidence intervals to compare relative validity coefficients: an example with PRO measures of chronic kidney disease (CKD) impact Value Health 2012 15 A159

[B48] Dorans NJ Equating, concordance, and expectation Applied Psychological Measurement 2004 28 227 246 10.1177/0146621604265031

[B49] McHorney CA Use of item response theory to link 3 modules of functional status items from the asset and health dynamics among the oldest old study Arch Phys Med Rehabil 2002 83 383 394 10.1053/apmr.2002.29610 11887121

[B50] Bjorner JB Kosinski M Ware JE Jr Using item response theory to calibrate the Headache Impact Test (HIT) to the metric of traditional headache scales Qual Life Res 2003 12 981 1002 10.1023/A:1026123400242 14651417

[B51] Holzner B Bode RK Hahn EA Cella D Kopp M Sperner-Unterweger B Kemmler G Equating EORTC QLQ-C30 and FACT-G scores and its use in oncological research Eur J Cancer 2006 42 3169 3177 10.1016/j.ejca.2006.08.016 17045472

[B52] Velozo CA Byers KL Wang YC Joseph BR Translating measures across the continuum of care: using Rasch analysis to create a crosswalk between the functional independence measure and the minimum data set J Rehabil Res Dev 2007 44 467 478 10.1682/JRRD.2006.06.0068 18247243

[B53] Haley SM Ni P Lai JS Tian F Coster WJ Jette AM Straub D Cella D Linking the activity measure for post acute care and the quality of life outcomes in neurological disorders Arch Phys Med Rehabil 2011 92 S37 S43 10.1016/j.apmr.2011.01.026 21958921 PMC3372982

[B54] Fischer HF Wahl I Fliege H Klapp BF Rose M Impact of cross-calibration methods on the interpretation of a treatment comparison study using 2 depression scales Med Care 2012 50 320 326 10.1097/MLR.0b013e31822945b4 22422054

[B55] Askew RL Kim J Chung H Cook KF Johnson KL Amtmann D Development of a crosswalk for pain interference measured by the BPI and PROMIS pain interference short form Qual Life Res 2013 (2013 Mar 29. [Epub ahead of print]) 10.1007/s11136-013-0398-5 23539469

[B56] Noonan VK Cook KF Bamer AM Choi SW Kim J Amtmann D Measuring fatigue in persons with multiple sclerosis: creating a crosswalk between the modified fatigue impact scale and the PROMIS fatigue short form Qual Life Res 2012 21 1123 1133 10.1007/s11136-011-0040-3 22048931

[B57] Fries JF Krishnan E Bruce B Items, instruments, crosswalks, and PROMIS J Rheumatol 2009 36 1093 1095 10.3899/jrheum.090320 19509084

[B58] Cella D Yount S Rothrock N Gershon R Cook K Reeve B Ader D Fries JF Bruce B Rose M The patient-reported outcomes measurement information system (PROMIS): progress of an NIH Roadmap cooperative group during its first two years Med Care 2007 45 S3 S11 17443116 10.1097/01.mlr.0000258615.42478.55 PMC2829758

[B59] Fries JF Cella D Rose M Krishnan E Bruce B Progress in assessing physical function in arthritis: PROMIS short forms and computerized adaptive testing J Rheumatol 2009 36 2061 2066 10.3899/jrheum.090358 19738214

[B60] Siemons L Ten Klooster PM Taal E Glas CA Van de Laar MA Modern psychometrics applied in rheumatology–A systematic review BMC Musculoskelet Disord 2012 13 216 10.1186/1471-2474-13-216 23114105 PMC3517453

[B61] Tennant A McKenna SP Hagell P Application of Rasch analysis in the development and application of quality of life instruments Value Health 2004 7 Suppl 1 S22 S26 15367240 10.1111/j.1524-4733.2004.7s106.x

[B62] Andrich D Controversy and the Rasch model: a characteristic of incompatible paradigms? Med Care 2004 42 I7 I16 14707751 10.1097/01.mlr.0000103528.48582.7c

[B63] Bond TG Fox CM Applying the Rasch model: Fundamental measurement in the human sciences 2007 Mahwah, NJ: Lawrence Erlbaum

[B64] Stucki G Stucki S Bruhlmann P Michel BA Ceiling effects of the health assessment questionnaire and its modified version in some ambulatory rheumatoid arthritis patients Ann Rheum Dis 1995 54 461 465 10.1136/ard.54.6.461 7632087 PMC1009903

[B65] Uhlig T Haavardsholm EA Kvien TK Comparison of the health assessment questionnaire (HAQ) and the modified HAQ (MHAQ) in patients with rheumatoid arthritis Rheumatology (Oxford) 2006 45 454 458 10.1093/rheumatology/kei181 16287925

[B66] Wolfe F Michaud K Pincus T Development and validation of the health assessment questionnaire II: a revised version of the health assessment questionnaire Arthritis Rheum 2004 50 3296 3305 10.1002/art.20549 15476213

[B67] Andresen EM Fouts BS Romeis JC Brownson CA Performance of health-related quality-of-life instruments in a spinal cord injured population Arch Phys Med Rehabil 1999 80 877 884 10.1016/S0003-9993(99)90077-1 10453762

[B68] Freeman JA Hobart JC Langdon DW Thompson AJ Clinical appropriateness: a key factor in outcome measure selection: the 36 item short form health survey in multiple sclerosis J Neurol Neurosurg Psychiatry 2000 68 150 156 10.1136/jnnp.68.2.150 10644779 PMC1736771

[B69] Kersten P Mullee MA Smith JA McLellan L George S Generic health status measures are unsuitable for measuring health status in severely disabled people Clin Rehabil 1999 13 219 228 10.1191/026921599667206154 10392649

[B70] Lai SM Perera S Duncan PW Bode R Physical and social functioning after stroke: comparison of the Stroke Impact Scale and Short Form-36 Stroke 2003 34 488 493 10.1161/01.STR.0000054162.94998.C0 12574565

[B71] Walsh M Macgregor D Stuckless S Barrett B Kawaja M Scully MF Health-related quality of life in a cohort of adult patients with mild hemophilia A J Thromb Haemost 2008 6 755 761 10.1111/j.1538-7836.2008.02929.x 18284605

[B72] Katz P Morris A Yelin E Subclinical disability in valued life activities among individuals with rheumatoid arthritis Arthritis Rheum 2008 59 1416 1423 10.1002/art.24110 18821642 PMC2754406

[B73] Langston AL Campbell MK Fraser WD Maclennan G Selby P Ralston SH Clinical determinants of quality of life in Paget’s disease of bone Calcif Tissue Int 2007 80 1 9 17205328 10.1007/s00223-006-0184-2

[B74] Lovas K Kalo Z McKenna SP Whalley D Pentek M Genti G Establishing a standard for patient-completed instrument adaptations in Eastern Europe: experience with the Nottingham health profile in Hungary Health Policy 2003 63 49 61 10.1016/S0168-8510(02)00078-7 12468117

[B75] Johnson SR Lee P The HAQ disability index in scleroderma trials Rheumatology (Oxford) 2004 43 1200 1201 10.1093/rheumatology/keh288 15317965

[B76] Zandbelt MM Welsing PM van Gestel AM van Riel PL Health assessment questionnaire modifications: is standardisation needed? Ann Rheum Dis 2001 60 841 845 11502610 PMC1753820

[B77] Wolfe F A reappraisal of HAQ disability in rheumatoid arthritis Arthritis Rheum 2000 43 2751 2761 10.1002/1529-0131(200012)43:12<2751::AID-ANR15>3.0.CO;2-6 11145033

[B78] Krishnan E Sokka T Hakkinen A Hubert H Hannonen P Normative values for the Health Assessment Questionnaire disability index: benchmarking disability in the general population Arthritis Rheum 2004 50 953 960 10.1002/art.20048 15022339

[B79] Louie GH Reveille JD Ward MM Challenges comparing functional limitations in rheumatoid arthritis and ankylosing spondylitis Clin Exp Rheumatol 2009 27 S83 S91 19822052 PMC2953760

[B80] Combe B Landewe R Lukas C Bolosiu HD Breedveld F Dougados M Emery P Ferraccioli G Hazes JM Klareskog L EULAR recommendations for the management of early arthritis: report of a task force of the European standing committee for international clinical studies including therapeutics (ESCISIT) Ann Rheum Dis 2007 66 34 45 16396980 10.1136/ard.2005.044354 PMC1798412

[B81] Smolen JS Aletaha D Bijlsma JW Breedveld FC Boumpas D Burmester G Combe B Cutolo M de Wit M Dougados M Treating rheumatoid arthritis to target: recommendations of an international task force Ann Rheum Dis 2010 69 631 637 10.1136/ard.2009.123919 20215140 PMC3015099

