# Supplementary material for: Development and evaluation of a crosswalk between the SF-36 physical functioning scale and Health Assessment Questionnaire disability index in rheumatoid arthritis
Source: Health Qual Life Outcomes. 2013 Nov 15;11:199. doi: 10.1186/1477-7525-11-199 (PMC3835476; doi:10.1186/1477-7525-11-199)
Supplement: Additional file 1: Table S1 — Item parameters and item level fit statistics for the Rasch (PCM) co-calibration of the HAQ-SDI and PF-10. Table S2. Item parameters and item level fit statistics for the Rasch (PCM) co-calibration of the HAQ-ADI and PF-10. Table S3. Item parameters and item level fit statistics for the two-parameter (GPCM) co-calibration of the HAQ-SDI and PF-10. Table S4. Item parameters and item level fit statistics for the two-parameter (GPCM) co-calibration of the HAQ-ADI and PF-10. Table S5. Item parameters and item level fit statistics for the multidimensional (GPCM) co-calibration of the HAQ-SDI and PF-10. Table S6. Item parameters and item level fit statistics for the multidimensional (GPCM) co-calibration of the HAQ-ADI and PF-10. [file 1477-7525-11-199-S1.docx]

Supplementary table 1. Item parameters and item level fit statistics for the Rasch (PCM) co-calibration of the HAQ-SDI and PF-10

|  | β_1_ (SD) | β_2_ (SD) | β_3_ (SD) | LM | p | ES |
| --- | --- | --- | --- | --- | --- | --- |
| HAQ-DI Dressing | -0.21 (0.08) | 1.44 (0.08) | 4.01 (0.17) | 7.03 | 0.03 | 0.03 |
| HAQ-DI Rising | -0.04 (0.07) | 1.81 (0.08) | 6.39 (0.39) | 12.18 | 0.00 | 0.03 |
| HAQ-DI Eating | -1.06 (0.08) | 1.22 (0.07) | 3.07 (0.11) | 6.15 | 0.05 | 0.03 |
| HAQ-DI Walking | 0.02 (0.08) | 1.51 (0.08) | 3.77 (0.15) | 9.53 | 0.01 | 0.03 |
| HAQ-DI Hygiene | -0.39 (0.09) | -0.52 (0.08) | 2.42 (0.08) | 26.43 | 0.00 | 0.06 |
| HAQ-DI Reaching | -0.68 (0.08) | 0.90 (0.08) | 3.12 (0.12) | 9.60 | 0.01 | 0.04 |
| HAQ-DI Grasping | -0.64 (0.09) | -0.90 (0.08) | 3.55 (0.12) | 42.21 | 0.00 | 0.08 |
| HAQ-DI Activities | -1.45 (0.09) | 0.11 (0.07) | 2.68 (0.10) | 13.72 | 0.00 | 0.04 |
| PF-10 Vigorous activities | -3.65 (0.13) | -1.12 (0.07) |  | 46.52 | 0.00 | 0.06 |
| PF-10 Moderate activities | -2.04 (0.10) | 1.55 (0.08) |  | 70.48 | 0.00 | 0.05 |
| PF-10 Lifting or carrying groceries | -1.99 (0.09) | 1.32 (0.08) |  | 9.35 | 0.01 | 0.02 |
| PF-10 Climbing several flights of stairs | -0.94 (0.08) | 1.40 (0.08) |  | 1.85 | 0.40 | 0.01 |
| PF-10 Climbing one flight of stairs | -0.13 (0.08) | 2.60 (0.09) |  | 14.17 | 0.00 | 0.03 |
| PF-10 Bending, kneeling or stooping | -1.86 (0.08) | 1.07 (0.07) |  | 0.95 | 0.62 | 0.01 |
| PF-10 Walking more than a mile | -0.84 (0.08) | 0.82 (0.08) |  | 0.46 | 0.79 | 0.01 |
| PF-10 Walking several blocks | 0.17 (0.10) | 2.12 (0.11) |  | 40.27 | 0.00 | 0.04 |
| PF-10 Walking one block | 0.69 (0.09) | 2.83 (0.13) |  | 38.96 | 0.00 | 0.03 |
| PF-10 Bathing and dressing self | 0.27 (0.08) | 3.62 (0.13) |  | 59.23 | 0.00 | 0.04 |

β = item difficulty threshold parameter; LM = Lagrange multiplier statistic; ES = effect size.

Supplementary table 2. Item parameters and item level fit statistics for the Rasch (PCM) co-calibration of the HAQ-ADI and PF-10

|  | β_1_ (SD) | β_2_ (SD) | β_3_ (SD) | LM | p | ES |
| --- | --- | --- | --- | --- | --- | --- |
| HAQ-DI Dressing | -0.23 (0.07) | 2.69 (0.10) | 3.62(0.18) | 22.35 | 0.00 | 0.03 |
| HAQ-DI Rising | -0.01 (0.07) | 2.82 (0.10) | 6.15 (0.41) | 16.38 | 0.00 | 0.03 |
| HAQ-DI Eating | -1.12 (0.08) | 1.52 (0.08) | 3.08 (0.11) | 17.94 | 0.00 | 0.04 |
| HAQ-DI Walking | -0.03 (0.12) | 2.41 (0.10) | 3.52 (0.16) | 17.96 | 0.00 | 0.03 |
| HAQ-DI Hygiene | -0.31 (0.08) | 1.79 (0.08) | 1.74 (0.10) | 16.71 | 0.00 | 0.06 |
| HAQ-DI Reaching | -0.82 (0.08) | 1.87 (0.08) | 2.88 (0.12) | 2.24 | 0.33 | 0.02 |
| HAQ-DI Grasping | -0.93 (0.07) | 1.78 (0.08) | 3.01 (0.12) | 26.50 | 0.00 | 0.05 |
| HAQ-DI Activities | -1.72 (0.09) | 1.39 (0.08) | 2.38 (0.11) | 33.62 | 0.00 | 0.05 |
| PF-10 Vigorous activities | -3.80 (0.13) | -1.17 (0.08) |  | 72.91 | 0.00 | 0.07 |
| PF-10 Moderate activities | -2.12 (0.10) | 1.62 (0.08) |  | 36.71 | 0.00 | 0.04 |
| PF-10 Lifting or carrying groceries | -2.07 (0.09) | 1.40 (0.08) |  | 1.43 | 0.49 | 0.01 |
| PF-10 Climbing several flights of stairs | -0.98 (0.08) | 1.48 (0.08) |  | 4.61 | 0.10 | 0.02 |
| PF-10 Climbing one flight of stairs | -0.12 (0.08) | 2.69 (0.10) |  | 10.08 | 0.01 | 0.03 |
| PF-10 Bending, kneeling or stooping | -1.93 (0.08) | 1.14 (0.07) |  | 4.73 | 0.09 | 0.02 |
| PF-10 Walking more than a mile | -0.88 (0.08) | 0.89 (0.08) |  | 2.91 | 0.23 | 0.01 |
| PF-10 Walking several blocks | 0.18 (0.10) | 2.21 (0.10) |  | 44.92 | 0.00 | 0.04 |
| PF-10 Walking one block | 0.73 (0.09) | 2.92 (0.12) |  | 67.19 | 0.00 | 0.04 |
| PF-10 Bathing and dressing self | 0.30 (0.08) | 3.70 (0.13) |  | 55.87 | 0.00 | 0.04 |

LM = Lagrange multiplier statistic; ES = Effect size; β = item difficulty threshold parameter (reflects the point on the latent scale where respondents have a 50% likelihood of choosing consecutive response options).

Supplementary table 3. Item parameters and item level fit statistics for the two-parameter (GPCM) co-calibration of the HAQ-SDI and PF-10

|  | α (SD) | β_1_ (SD) | β_2_ (SD) | β_3_ (SD) | LM | p | ES |
| --- | --- | --- | --- | --- | --- | --- | --- |
| HAQ-DI Dressing | 1.69 (0.09) | -0.11 (0.08) | 0.83 (0.08) | 2.30 (0.10) | 9.47 | 0.01 | 0.03 |
| HAQ-DI Rising | 1.80 (0.09) | -0.02 (0.25) | 1.02 (0.08) | 3.60 (0.07) | 15.63 | 0.00 | 0.03 |
| HAQ-DI Eating | 1.38 (0.07) | -0.63 (0.11) | 0.75 (0.48) | 1.84 (0.07) | 16.01 | 0.00 | 0.03 |
| HAQ-DI Walking | 2.11 (0.11) | -0.00 (0.08) | 0.84 (0.10) | 2.03 (0.17) | 0.03 | 0.98 | 0.00 |
| HAQ-DI Hygiene | 1.15 (0.06) | -0.01 (0.10) | -0.54 (0.08) | 1.59 (0.12) | 6.52 | 0.04 | 0.02 |
| HAQ-DI Reaching | 1.46 (0.07) | -0.37 (0.29) | 0.53 (0.05) | 1.86 (0.09) | 19.24 | 0.00 | 0.04 |
| HAQ-DI Grasping | 1.10 (0.06) | -0.13 (0.09) | -0.89 (0.11) | 2.54 (0.07) | 2.25 | 0.33 | 0.01 |
| HAQ-DI Activities | 1.97 (0.09) | -0.82 (0.08) | 0.08 (0.09) | 1.46 (0.16) | 6.35 | 0.04 | 0.03 |
| PF-10 Vigorous activities | 1.19 (0.07) | -2.32 (0.05) | -0.88 (0.09) |  | 5.92 | 0.05 | 0.01 |
| PF-10 Moderate activities | 2.61 (0.13) | -1.04 (0.09) | 0.79 (0.14) |  | 2.06 | 0.36 | 0.01 |
| PF-10 Lifting or carrying groceries | 2.16 (0.11) | -1.07 (0.09) | 0.71 (0.11) |  | 2.10 | 0.35 | 0.01 |
| PF-10 Climbing several flights of stairs | 1.94 (0.11) | -0.52 (0.12) | 0.78 (0.16) |  | 1.70 | 0.43 | 0.01 |
| PF-10 Climbing one flight of stairs | 2.27 (0.11) | -0.07 (0.06) | 1.37 (0.13) |  | 4.24 | 0.12 | 0.01 |
| PF-10 Bending, kneeling or stooping | 1.70 (0.08) | -1.07 (0.14) | 0.61 (0.13) |  | 1.91 | 0.38 | 0.01 |
| PF-10 Walking more than a mile | 1.84 (0.10) | -0.47 (0.14) | 0.47 (0.14) |  | 5.04 | 0.08 | 0.01 |
| PF-10 Walking several blocks | 2.50 (0.15) | 0.06 (0.11) | 1.13 (0.12) |  | 0.80 | 0.67 | 0.01 |
| PF-10 Walking one block | 2.26 (0.14) | 0.34 (0.13) | 1.50 (0.10) |  | 8.44 | 0.01 | 0.02 |
| PF-10 Bathing and dressing self | 2.26 (0.12) | 0.14 (0.09) | 1.88 (0.12) |  | 17.48 | 0.00 | 0.02 |

α = item discrimination parameter; β = item difficulty threshold parameter; LM = Lagrange multiplier statistic; ES = effect size.

Supplementary table 4. Item parameters and item level fit statistics for the two-parameter (GPCM) co-calibration of the HAQ-ADI and PF-10

|  | α (SD) | β_1_ (SD) | β_2_ (SD) | β_3_ (SD) | LM | p | ES |
| --- | --- | --- | --- | --- | --- | --- | --- |
| HAQ-DI Dressing | 2.15 (0.11) | -0.14 (0.11) | 1.36 (0.08) | 1.93 (0.15) | 8.34 | 0.02 | 0.02 |
| HAQ-DI Rising | 2.00 (0.10) | -0.02 (0.31) | 1.47 (0.10) | 3.28 (0.07) | 10.97 | 0.00 | 0.02 |
| HAQ-DI Eating | 1.42 (0.07) | -0.66 (0.15) | 0.89 (0.53) | 1.74 (0.07) | 2.47 | 0.29 | 0.01 |
| HAQ-DI Walking | 2.21 (0.11) | -0.04 (0.08) | 1.22 (0.10) | 1.84 (0.18) | 2.74 | 0.25 | 0.01 |
| HAQ-DI Hygiene | 1.31 (0.05) | -0.14 (0.11) | 1.12 (0.08) | 0.84 (0.14) | 7.52 | 0.02 | 0.03 |
| HAQ-DI Reaching | 1.55 (0.07) | -0.47 (0.32) | 1.07 (0.05) | 1.58 (0.07) | 7.13 | 0.03 | 0.02 |
| HAQ-DI Grasping | 1.28 (0.06) | -0.56 (0.10) | 1.10 (0.12) | 1.72 (0.07) | 3.23 | 0.20 | 0.01 |
| HAQ-DI Activities | 2.35 (0.11) | -0.90 (0.08) | 0.67 (0.10) | 1.25 (0.17) | 3.25 | 0.20 | 0.01 |
| PF-10 Vigorous activities | 1.16 (0.06) | -2.37 (0.06) | -0.91 (0.07) |  | 7.27 | 0.03 | 0.01 |
| PF-10 Moderate activities | 2.55 (0.12) | -1.07 (0.10) | 0.79 (0.17) |  | 4.25 | 0.12 | 0.01 |
| PF-10 Lifting or carrying groceries | 2.08 (0.10) | -1.11 (0.11) | 0.72 (0.12) |  | 3.54 | 0.17 | 0.01 |
| PF-10 Climbing several flights of stairs | 1.87 (0.10) | -0.55 (0.13) | 0.79 (0.18) |  | 6.92 | 0.03 | 0.02 |
| PF-10 Climbing one flight of stairs | 2.22 (0.11) | -0.08 (0.06) | 1.37 (0.13) |  | 12.03 | 0.00 | 0.03 |
| PF-10 Bending, kneeling or stooping | 1.66 (0.08) | -1.10 (0.15) | 0.61 (0.12) |  | 1.36 | 0.51 | 0.01 |
| PF-10 Walking more than a mile | 1.79 (0.09) | -0.49 (0.14) | 0.47 (0.14) |  | 2.62 | 0.27 | 0.01 |
| PF-10 Walking several blocks | 2.40 (0.14) | 0.05 (0.10) | 1.13 (0.12) |  | 8.52 | 0.01 | 0.02 |
| PF-10 Walking one block | 2.24 (0.14) | 0.34 (0.13) | 1.50 (0.10) |  | 31.34 | 0.00 | 0.02 |
| PF-10 Bathing and dressing self | 2.39 (0.12) | 0.13 (0.09) | 1.84 (0.11) |  | 14.80 | 0.00 | 0.02 |

α = item discrimination parameter; β = item difficulty threshold parameter; LM = Lagrange multiplier statistic; ES = effect size.

Supplementary table 5. Item parameters and item level fit statistics for the multidimensional (GPCM) co-calibration of the HAQ-SDI and PF-10

|  | α (SD) | β_1_ (SD) | β_2_ (SD) | β_3_ (SD) | LM | p | ES |
| --- | --- | --- | --- | --- | --- | --- | --- |
| HAQ-DI Dressing | 1.44 (0.07) | -0.54 (0.00) | 1.29 (0.00) | 3.94 (0.01) | 3.85 | 0.15 | 0.01 |
| HAQ-DI Rising | 1.42 (0.07) | -0.34 (0.06) | 1.66 (0.00) | 6.14 (0.00) | 28.72 | 0.00 | 0.03 |
| HAQ-DI Eating | 1.34 (0.07) | -1.33 (0.01) | 0.97 (0.20) | 2.84 (0.00) | 1.20 | 0.55 | 0.01 |
| HAQ-DI Walking | 1.22 (0.06) | -0.19 (0.00) | 1.19 (0.01) | 3.27 (0.03) | 84.21 | 0.00 | 0.06 |
| HAQ-DI Hygiene | 0.99 (0.05) | -0.30 (0.00) | -0.81 (0.00) | 1.80 (0.01) | 9.86 | 0.01 | 0.04 |
| HAQ-DI Reaching | 1.44 (0.07) | -1.02 (0.05) | 0.69 (0.00) | 3.05 (0.01) | 3.02 | 0.22 | 0.02 |
| HAQ-DI Grasping | 1.05 (0.05) | -0.55 (0.01) | -1.16 (0.01) | 2.91 (0.00) | 28.96 | 0.00 | 0.04 |
| HAQ-DI Activities | 1.64 (0.08) | -2.06 (0.00) | -0.16 (0.01) | 2.83 (0.02) | 32.72 | 0.00 | 0.06 |
| PF-10 Vigorous activities | 0.97 (0.05) | -2.93 (0.00) | -1.25 (0.01) |  | 5.85 | 0.05 | 0.01 |
| PF-10 Moderate activities | 1.82 (0.10) | -2.82 (0.01) | 1.57 (0.02) |  | 39.93 | 0.00 | 0.02 |
| PF-10 Lifting or carrying groceries | 1.50 (0.08) | -2.40 (0.00) | 1.14 (0.01) |  | 24.61 | 0.00 | 0.02 |
| PF-10 Climbing several flights of stairs | 1.72 (0.10) | -1.50 (0.02) | 1.40 (0.02) |  | 2.29 | 0.32 | 0.01 |
| PF-10 Climbing one flight of stairs | 2.02 (0.11) | -0.62 (0.00) | 3.15 (0.01) |  | 0.69 | 0.71 | 0.00 |
| PF-10 Bending, kneeling or stooping | 1.34 (0.07) | -2.08 (0.02) | 0.81 (0.01) |  | 10.26 | 0.01 | 0.02 |
| PF-10 Walking more than a mile | 1.79 (0.10) | -1.47 (0.02) | 0.77 (0.02) |  | 24.04 | 0.00 | 0.02 |
| PF-10 Walking several blocks | 2.69 (0.17) | -0.43 (0.00) | 3.36 (0.01) |  | 8.86 | 0.01 | 0.01 |
| PF-10 Walking one block | 2.22 (0.13) | 0.43 (0.01) | 3.80 (0.01) |  | 9.65 | 0.01 | 0.02 |
| PF-10 Bathing and dressing self | 1.51 (0.09) | -0.00 (0.01) | 3.59 (0.01) |  | 89.55 | 0.00 | 0.04 |

α = item discrimination parameter; β = item difficulty threshold parameter; LM = Lagrange multiplier statistic; ES = effect size.

Supplementary table 6. Item parameters and item level fit statistics for the multidimensional (GPCM) co-calibration of the HAQ-ADI and PF-10

|  | α (SD) | β_1_ (SD) | β_2_ (SD) | β_3_ (SD) | LM | p | ES |
| --- | --- | --- | --- | --- | --- | --- | --- |
| HAQ-DI Dressing | 1.88 (0.10) | -0.70 (0.01) | 3.00 (0.01) | 4.38 (0.02) | 6.96 | 0.03 | 0.02 |
| HAQ-DI Rising | 1.58 (0.08) | -0.33 (0.10) | 2.83 (0.00) | 6.20 (0.00) | 21.38 | 0.00 | 0.02 |
| HAQ-DI Eating | 1.33 (0.06) | -1.34 (0.02) | 1.23 (0.25) | 2.74 (0.00) | 14.86 | 0.00 | 0.02 |
| HAQ-DI Walking | 1.37 (0.07) | -0.30 (0.00) | 2.16 (0.01) | 3.23 (0.03) | 74.83 | 0.00 | 0.04 |
| HAQ-DI Hygiene | 1.04 (0.05) | -0.38 (0.00) | 1.34 (0.00) | 1.04 (0.01) | 11.22 | 0.00 | 0.03 |
| HAQ-DI Reaching | 1.40 (0.07) | -1.10 (0.06) | 1.64 (0.00) | 2.67 (0.00) | 0.26 | 0.88 | 0.00 |
| HAQ-DI Grasping | 1.16 (0.06) | -1.02 (0.01) | 1.39 (0.01) | 2.40 (0.00) | 2.62 | 0.27 | 0.01 |
| HAQ-DI Activities | 2.01 (0.10) | -2.64 (0.00) | 1.37 (0.01) | 3.07 (0.03) | 6.44 | 0.04 | 0.01 |
| PF-10 Vigorous activities | 0.97 (0.05) | -2.93 (0.00) | -1.24 (0.00) |  | 8.93 | 0.01 | 0.01 |
| PF-10 Moderate activities | 1.77 (0.10) | -2.78 (0.01) | 1.59 (0.02) |  | 28.40 | 0.00 | 0.02 |
| PF-10 Lifting or carrying groceries | 1.47 (0.08) | -2.38 (0.01) | 1.16 (0.02) |  | 13.11 | 0.00 | 0.01 |
| PF-10 Climbing several flights of stairs | 1.70 (0.09) | -1.49 (0.02) | 1.43 (0.03) |  | 15.02 | 0.00 | 0.02 |
| PF-10 Climbing one flight of stairs | 1.96 (0.10) | -0.60 (0.00) | 3.15 (0.01) |  | 2.91 | 0.23 | 0.01 |
| PF-10 Bending, kneeling or stooping | 1.32 (0.07) | -2.07 (0.02) | 0.82 (0.01) |  | 4.48 | 0.11 | 0.01 |
| PF-10 Walking more than a mile | 1.80 (0.10) | -1.50 (0.02) | 0.82 (0.02) |  | 57.29 | 0.00 | 0.03 |
| PF-10 Walking several blocks | 2.60 (0.16) | -0.41 (0.00) | 3.36 (0.01) |  | 2.90 | 0.23 | 0.01 |
| PF-10 Walking one block | 2.08 (0.13) | 0.45 (0.01) | 3.70 (0.01) |  | 11.68 | 0.00 | 0.01 |
| PF-10 Bathing and dressing self | 1.45 (0.08) | 0.00 (0.01) | 3.56 (0.01) |  | 24.16 | 0.00 | 0.05 |

α = item discrimination parameter; β = item difficulty threshold parameter; LM = Lagrange multiplier statistic; ES = effect size.
